# Enhancing Early Detection of Neurodegenerative Disease Through Advanced Verbal Fluency Analysis

**DOI:** 10.1002/alz70857_102681

**Published:** 2025-12-25

**Authors:** Alice Stanton, Claire L Lancaster, Jennifer M Rusted, Naji Tabet

**Affiliations:** ^1^ University of Sussex, Brighton, East Sussex, United Kingdom; ^2^ Brighton & Sussex Medical School, Brighton, United Kingdom; ^3^ University of Sussex, Brighton, United Kingdom; ^4^ Brighton & Sussex Medical School, Brighton, East Sussex, United Kingdom

## Abstract

**Background:**

Early detection of neurodegenerative disease is crucial. Traditional Verbal Fluency Task (VFT) scoring may miss subtle preclinical neurocognitive changes. This systematic review evaluated nuanced manual and automated VFT scoring methods, including advanced clustering and switching metrics, for identifying individuals at risk of future dementia.

**Methods:**

A systematic search of PubMed, PsycINFO, and Scopus (inception to Sept 2024; PRISMA guidelines) was conducted. Key inclusion criteria were: 1) use of automated or non‐traditional manual scoring of semantic and phonemic verbal fluency; 2) VFT completed by adults with Alzheimer's disease (AD), Mild Cognitive Impairment (MCI), AD biomarker positivity, and/or carrying an APOE4 genetic risk variant. Studies focusing solely on traditional scoring (total words produced) or acoustic speech features were excluded. Findings were synthesized narratively.

**Results:**

Fifty‐three studies met criteria (41.5% automated, 58.5% novel manual). Advanced quantification of semantic clustering (Word2Vec, t‐SNE) aided detection of early cognitive impairment by revealing subtle differences in semantic memory. Longitudinal studies showed enhanced predictive power ‐ for example, incorporating automated clustering metrics improved diagnostic reclassification by 5 percentage points compared to traditional scoring over a 9‐year period. Integrating linguistic features like word frequency and semantic “typicality” enhanced diagnostic accuracy, with increased word typicality associated with elevated risk of transitioning from MCI to AD. Temporal dynamics of lexical switching capture early executive dysfunction missed by traditional scoring.

**Conclusions:**

Both automated and novel manual VFT analyses show promise for detecting early cognitive decline. Automated methods offer advantages in processing speed and consistency, while manual approaches provide clinical interpretability and flexibility. Future work should focus on developing transparent, validated automated tools through clinician co‐design, with emphasis on utility across diverse populations and seamless integration into clinical workflows.

